# Retro-Element *Gypsy-*163 Is Differentially Methylated in Reproductive Tissues of Apomictic and Sexual Plants of *Cenchrus ciliaris*

**DOI:** 10.3389/fgene.2020.00795

**Published:** 2020-07-22

**Authors:** Priyanka Rathore, Soom Nath Raina, Suresh Kumar, Vishnu Bhat

**Affiliations:** ^1^Department of Botany, Faculty of Science, University of Delhi, New Delhi, India; ^2^Amity Institute of Biotechnology, Amity University, Noida, India; ^3^Division of Biochemistry, ICAR-Indian Agricultural Research Institute, New Delhi, India

**Keywords:** apomixis, *Cenchrus ciliaris*, Gypsy, DNA methylation, epigenetics, mode of reproduction, retro-element

## Abstract

Apomixis, an asexual mode of reproduction through seeds, has immense scope for crop improvement due to its ability to fix hybrid vigor. In *C. ciliaris*, a predominantly apomictically reproducing range grass, apomixis is genetically controlled by an apospory-specific-genomic-region (ASGR) which is enriched with retrotransposons. Earlier studies showed insertional polymorphisms of a few ASGR-specific retrotransposons between apomictic and sexual plants of *C. ciliaris*. REs are mainly regulated at the transcriptional level through cytosine methylation. To understand the possible association of ASGR-specific retrotransposon to apomixis, the extent and pattern of differential methylation of *Gy*163 RE and its impact on transcription were investigated in two genotypes each of apomictic and sexual plants of *C. ciliaris*. We observed that *Gy*163 encodes for an integrase domain of RE Ty3-*Gypsy*, is differentially methylated between reproductive tissues of apomictic and sexual plants. However, leaf tissues did not exhibit differential methylation between apomictic and sexual plants. Among the three contexts (CG, CHG, and CHH) of cytosine methylation, the maximum variation was observed in CHH context in reproductive (at aposporous initial and mature embryo sac stages) tissues of apomictic plants implicating RdDM pathway in methylation of *Gy*163. Quantitative PCR analysis showed that *Gy*163 transcripts are expressed more in the reproductive tissues of apomictic plants compared to that in the sexual plants, which was negatively correlated with the methylation level. Thus, the study helps in understanding the role of RE present in ASGR in epigenetic regulation of apomictic mode of reproduction in *C. ciliaris*.

## Introduction

Apomixis is an asexual mode of reproduction through seeds which bypasses meiotic division and fertilization of egg cell to produce progenies that are the replica of the mother plant (Kumar, 2017). Apomixis possesses a significant role in crop improvement because of its potential role in fixing heterosis and facilitating hybrid seeds production without the need of maintaining parental inbred lines (Koltunow and Tucker, 2003; Kumar et al., 2019). In crops like maize and rice, harnessing apomixis might lead to a great impact on hybrid seed production as it would lower the cost of seed production (Spillane et al., 2001; Kumar and Bhat, 2013). The traditional breeding approach for incorporating apomixis in crop plants through interspecific hybridization resulted in the production of unviable germplasm (Bicknell and Koltunow, 2004). Therefore, efforts are being made to harness apomixis through molecular genetics by identifying the key genes associated with regulating apomixis in plants (Bicknell and Koltunow, 2004; Singh et al., 2007; Kumar, 2019). Over 400 genera including Asteraceae, Rosaceae, and Poaceae reproduce through apomixis (Carman, 1997; Hojsgaard et al., 2014). Apomixis is considered to be a variation in sexual reproduction that includes other structural forms of female gametogenesis such as diplospory and polyembryony. Mode of reproduction is regulated through genetic and epigenetic mechanisms (Kawashima and Berger, 2014). The genetic and molecular basis of apomixis is still not well-known (Kumar, 2017). Although different models have been proposed for explaining the sexual and apomictic modes of reproduction, yet successful transfer of apomixis to a crop plant is still awaited (Hörandl and Hojsgaard, 2012; Kumar et al., 2019). Recent evidence shows that female gametophyte development and seed formation are controlled by epigenetic mechanisms that distinguish sexual and apomictic development (Rodríguez-Leal et al., 2015). The large majority of apomictic genotypes are polyploid which suggests that apomixis could have evolved from hybridization followed by polyploidization (Quarin et al., 2001; Matzk et al., 2003; Hörandl and Hojsgaard, 2012). Recently, it is also proposed that apomixis might be regulated through epigenetic mechanisms (Rodrigues and Koltunow, 2005; Curtis and Grossniklaus, 2008; Podio et al., 2012, 2014; Zappacosta et al., 2014; Kumar et al., 2017b; Bocchini et al., 2018; Albertini et al., 2019). Many reports showed that mutation in ARGONAUTE 9 (AGO9), which is involved in methylation of retroelements (REs) in the female germline, results in apomixis like trait in maize and *Arabidopsis* (Olmedo-Monfil et al., 2010; Singh et al., 2011).

**FIGURE 1:**
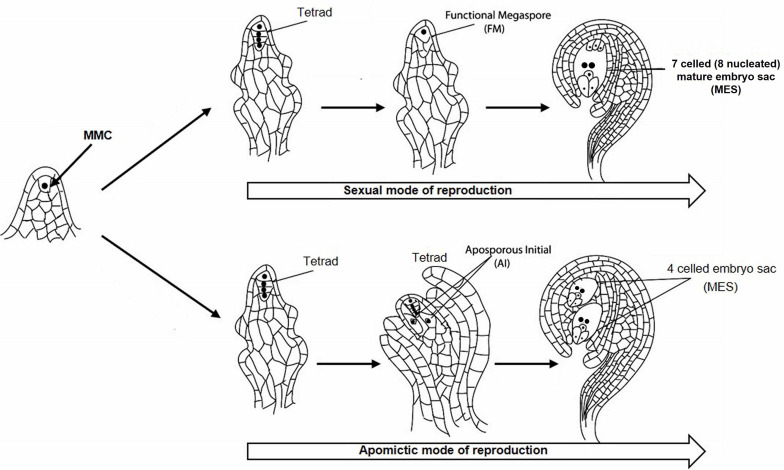
Sexual and apomictic mode of reproduction starting from megaspore mother cell (MMC) in the plant. MMC divides to generate a tetrad structure, which proceeds toward the development of reduced functional megaspore (FM) followed by the formation of 7 celled mature embryo sac (MES) through the sexual mode of reproduction. The tetrad may take an apomictic mode of reproduction through the formation of aposporous initial (AI) followed by the development of 4 celled unreduced MES.

*Cenchrus ciliaris*, a perennial apomictic forage grass, belongs to the Poaceae family and distributed throughout tropical and temperate zones mainly in Africa, Asia, and Australia. *C. ciliaris* is cross-pollinated and allotetraploid with chromosome number 2*n* = 4 × = 36 (Akiyama et al., 2005; Kumar et al., 2015). *C. ciliaris* reproduces predominantly by apomixis, although tetraploid sexual lines have also been reported (Bray, 1978; Kumar et al., 2013). During sexual reproduction, megaspore mother cell (MMC) undergoes meiosis to form tetrad haploid megaspores, out of whichonly one functional megaspore gives rise to a seven-celled embryo sac (Figure 1). In *C. ciliaris*, gametophytic apomixis is found in which a diploid aposporous initial (AI) originates from nucellar tissues around the MMC which produce an unreduced aposporous embryo sac (Koltunow, 1993; Chen et al., 2000; Ross et al., 2004). AI may completely displace sexual structures, or sexual development may persist in the same ovule along with the apomictic development. Unlike sexual gametophyte development, multiple AIs may exist in a single ovule, which results in the formation of multiple apomictic embryo sacs.

Apomixis is reported to be genetically controlled by the apospory-specific genomic region (ASGR) in *C. ciliaris* (Roche et al., 1999; Ozias-Akins et al., 2003). ASGR is a dominant, hemizygous, heterochromatic region with suppressed recombination, rich in repetitive sequences such as retrotransposons (Akiyama et al., 2005; Calderini et al., 2006; Conner et al., 2008). Many earlier reports suggest that some of the transcripts from retrotransposons are differentially expressed between the apomictic and sexual plants (Rodrigues et al., 2003; Laspina et al., 2008; Ochogavıa et al., 2011; Yadav, 2011). Retrotransposons play important roles in eukaryotes, as they act as a driving force in the processes like mutation, recombination, and genome stability (Le et al., 2015; Kumar, 2018). Retrotransposons, class I transposable elements (TEs), are one of the major sources of genetic diversity, because of their mobility they contribute to increase the genome size in plants (Kumar and Bennetzen, 2000). Retrotransposon contains at least two important open reading frames (ORFs): *Gag* ORF and a polymerase (*Pol*) ORF for the enzymes which help in replication and integration of the RE in the host plant genome. While *Gag* is responsible for the packaging of retroelements (REs), *Pol* encodes for protease (PR), reverse transcriptase (RT), integrase (INT), and RNase H, which are essential for amplification and integration of the RE in plant genome (Suoniemi et al., 1998). REs are categorized into two groups (i) long terminal repeats REs (LTRs), and (ii) non-LTR REs based on their flanking terminal repeats (Sormacheva and Blinov, 2011). LTR REs are further sub-classified into Ty1-*Copia* (*Pseudoviridae)* and Ty3-*Gypsy* (*Metaviridae)* based on the order of RT and INT domains present in *Pol*, and the extent of similarity in their sequences (Kumar and Bennetzen, 1999).

Transposition of RE is a highly regulated phenomenon to avoid their deleterious effects on the host genome. Epigenetic mechanisms have been reported to control the movement of RE in the genome (Slotkin and Martienssen, 2007; Fedoroff, 2012). DNA methylation is an important epigenetic phenomenon that usually occurs at the 5th carbon of cytosine residue. DNA methylation also plays important roles in many biological processes such as growth and development by regulating gene expression and in genome stability (Saze et al., 2012; Lang et al., 2017; Kumar et al., 2018). Heterochromatin in eukaryotes is a highly condensed, hypermethylated, and recombinationally inert chromosomal region which is also rich in repetitive DNA and TEs (Fedoroff, 2012). In plants, cytosine methylation may occur in all three cytosine contexts (CG, CHG, and CHH, where H = A, T, or C) (Wang et al., 2016). Methylation of cytosine at CHH context is a common feature in REs, and it is a primary target for epigenetic silencing to inactivate them in plants, which gets reactivated in methylase deficient individuals resulting in accumulation of REs (Miura et al., 2001). Rabinowicz et al. (2003) reported a high level of genomic methylation mainly in TEs and repeats. Studies report activation of REs under stress, different developmental stage, and reproductive phase (Grandbastien, 1998; Beguiristain et al., 2001; McCue et al., 2012).

Alignment of *C. ciliaris* GSS/BAC sequences (GenBank: ED546266.1–ED544199.1, Conner et al., 2008) by Yadav (2011) resulted in the preparation of a few contigs. Based on the analysis of these contigs using repeat junction marker (RJM) strategy, some of the contigs, including *Gy*163, were reported to be polymorphic between apomictic and sexual *C. ciliaris* plants. In RJM strategy, the insertion of REs creates a unique junction site in ASGR therefore, one primer is designed from flanking inserted RE and target genomic sequence and another primer from the genomic or repetitive sequence (Frank et al., 2010). Using the insertional polymorphism approach, some of the REs present in the ASGR of apomictic *C. ciliaris* were identified (Yadav, 2011), and many of these REs, including *Gy*163, were observed to be polymorphic between apomictic and sexual *C. ciliaris* plants. In this study, we aimed at comparing the DNA methylation status of specific regions of RE *Gy*163 between apomictic and sexual plants of *C. ciliaris.* It also aims to investigate whether differentially methylated REs are associated with the mode of reproduction in *C. ciliaris*, and to study the correlation between DNA methylation and gene expression. This study helps to understand the epigenetic regulation of apomixis in *C. ciliaris*.

## Materials and Methods

### Plant Materials

Two obligate apomictic (CcApo7-5, CcApo18-2) and two obligate sexual (CcSex16-5, CcSex2-2) individuals of the F_2_ population of *C. ciliaris* (2*n* = 4× = 36) were used in this study. The F_2_ population was obtained by crossing obligate apomictic as male parent and obligate sexual as a female parent (Yadav et al., 2012). Two stages of embryo sac development were analyzed in this study i.e., AI and MES which represent two important developmental events apomeiosis and parthenogenesis during apospory in *C. ciliaris*, respectively. Different developmental stages of embryo sac development of *C. ciliaris* were identified according to Sharma et al. (2014). Genomic DNA (gDNA) was isolated from leaf, florets [at functional megaspore (FM) stage in case of sexual plants or AI stage in case of apomictic plants], and pistils of the mature embryo sac (MES) of sexual and apomictic plants using DNeasy Plant Mini Kit (Qiagen). Pistils and florets were dissected out from the inflorescence with the help of very fine tip forceps and needles under the Zeiss streomicroscope (Stemi DV4). The quality of the isolated gDNA was analyzed during agarose gel (0.8%) electrophoresis and spectrophotometry (Thermo Scientific Multiskan GO spectrophotometer).

### Characterization of Inserted/Conserved RE Regions in ASGR

**FIGURE 2:**
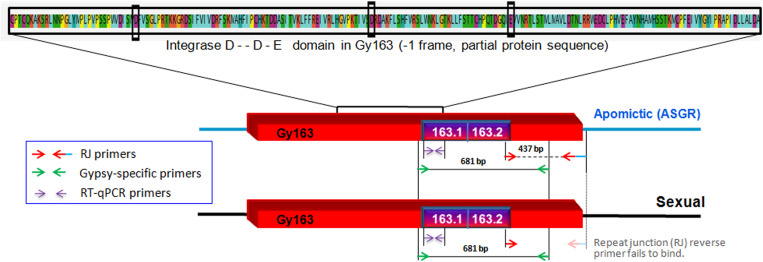
Diagrammatic representation of inserted/conserved retrotransposon (*Gy*163) in the apospory-specific genomic region (ASGR) of apomictic and in the sexual genome. Repeat junction (RJ) primers showing polymorphism between apomictic and sexual individuals, and *Gypsy*-specific primers showing a conserved region of *Gy*163 between apomictic and sexual plants. Different parts of *Gy*163 were used for DNA methylation (163.1 and 163.2), gene (integrase) expression, and polymorphism (RJ) analyses. The red box indicates the *Gy*163 retrotransposon, the blue line represents ASGR, and the blackline represents its counterpart in the sexual genome.

**FIGURE 3:**
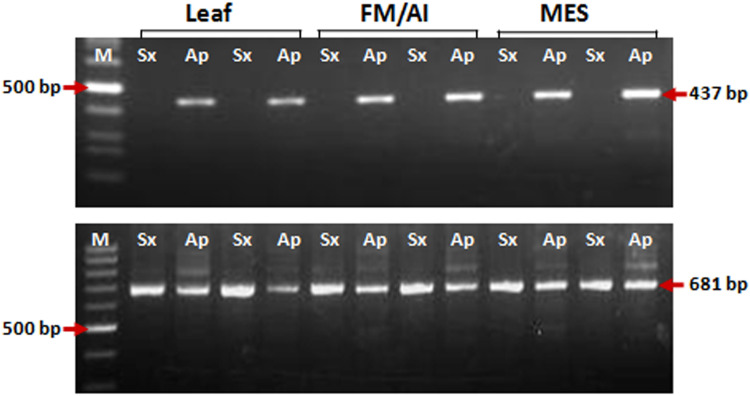
PCR amplification for insertional characterization of *Gy*163 in different tissues [leaf, non-reproductive; functional megaspore (FM in the sexual plant) or aposporous initial (AI in the apomictic plant) and mature embryo sac (MES), reproductive tissues] of sexual and apomictic plants. **(A)** RJ primer-based insertional polymorphism of *Gy*163 between apomictic (Ap) and sexual (Sx) plants, **(B)** Conserved *Gypsy* (*Gy*163)-specific primers showed a monomorphic band in different tissues of apomictic and sexual plants.

Contig 163, earlier reported to be polymorphic between apomictic and sexual plants of *C. ciliaris* based on the repeat junction (RJ) and expression analysis (Conner et al., 2008; Yadav, 2011), was used for DNA methylation analysis in the present study (BAC clone: C018A_58_C11.b1_A013 C018; GenBank: ED544700.1). The junction region was identified using RJ primer software^1^. A new set of primers were designed manually from the junction region of the inserted RE and the apomictic genomic region (Table 1) to further characterize and validate *Gy*163 insertion in ASGR. While the forward primer was designed from the RE sequence, the reverse primer was designed from the junction region of RE and genomic sequences (Figure 2). Different regions and domains of the RE were identified using RepeatMasker^2^ and RepeatExplorer^3^. As a counterpart of the RE is also present in sexual plants, primers were designed from the conserved region of Ty3-*gypsy* RE (conserved *gypsy*-specific primers, Table 1) to identify the corresponding region in the sexual plants, which was used for comparing methylation status of the RE in apomictic and sexual plants. In our study, we first amplified *Gy*163 from apomictic and sexual plants and checked/validated the sequence which showed >95% similarity with the partial/fragmented sequences of ASGR available in the NCBI database. The observed variability (up to 5%) in the retro-element could be due to the demographic variability between the *C. ciliaris* plants in the discussion. More than 470 bp of *Gy*163 (*Gy*163.1 and *Gy*163.2) were used to check methylation status in the regions and its effect on the expression of the gene. RJ primer-based insertional polymorphism of *Gy*163 between apomictic and sexual plants (Figure 3) indicates its location in the ASGR.

**TABLE 1:** List of primers used for polymorphism, epigenetic and expression analyses of *Gy*163.

S. no.	Primers		Primer mame	Primer sequence (5′→3′)	Annealing temp. (°C)	Amplicon size (bp)
1	Repeat Junction primers		GY163RJ_F	TTGAGAGCATGGTATATCGACGAGA	58	437
			GY163RJ_R	GATAATAATAATTTACCTGAACGAT		
2	Conserved gypsy-specific primers		GY163IN_F	AATGCTGTCCCTCCCTTTC	55	681
			GY163IN_R	GCTCTAGTTCGCACTTTG		
3	Bisulfite primers	Primary primers	GY163.1BSP1_F	TTYTTAGTTYTAGGYAATYYYGAA	55	282
			GY163.1BSP1_R	TRTTCCARCAARCCTRRTTCATCT		
			GY163.2BSP1_F	TGAGGYYGYYAYYATGTGYTTYYTG	55	252
			GY163.2BSP1_R	TTRCTRATRCTTTRTCTCRTCRAT		
		Secondary primers	GY163.1BSP2_F	YGAAAYAAAGTYYATAGAAATA	55	251
			GY163.1BSP2_R	RCCTRRTTCATCTTTTRCTRTT		
			GY163.2BSP2_F	TYYTGYAAYAGYAAAAGATGAAY	55	219
			GY163.2BSP2_R	RTCTCRTCRATATACCATRCTCTC		
4		RT-qPCR primers	GY163.1RT_F	CATAGAAATATCAACCCAAGGAGAA	60	83
			GY163.1RT_R	AGAAAGCTAAGTCGCGTTTGAA		
			Actin_F	CCTTCCTGATATCCACATCACA	60	103
			Actin_R	CCTGAGGTCCTCTTCCAACC		

*Where Y = C or T; R = A or G.*

### Bisulfite Sequencing

Methylation status of *Gy*163 in different tissues [leaf, florets (AI/FM) and pistils (MES)] of apomictic and sexually reproducing plants was analyzed using bisulfite sequencing (BS) to detect cytosine methylation. Bisulfite modification of gDNA was performed using BisulFlash DNA modification kit (Epigentek, Brooklyn, NY, United States) following the manufacturer’s instructions. From the bisulfite modified gDNA, two constitutive, conserved, common regions of the RE (*Gy*163.1 and *Gy*163.2) were PCR amplified using two bisulfite-specific nested primer pairs (Table 1). The PCR products were cloned in pGEM–T Easy vector (Promega) and 10 random clones for each sample tissue were sequenced. Further, PCR amplified products from the bisulfite converted template DNA were checked for all the four nucleotides in addition to the expected C to T conversion. Moreover, we used 2 plants of each (apomictic and sexual) type, 3 different tissues, and 2 fragments of the retro-element. The replicated experimentation (2 plants of each sexual and apomictic types, 3 different tissues, and sequencing of 10 colonies at 5× depth) is considered to have generated >60× (uniform) data in total, which provides sufficient confidence for the amplified retro-element and its location in the ASGR. For comparative analysis, the sequences for different tissues and modes of reproduction were aligned using ClustalX software and visualized manually using BioEdit graphical view (Srivastava et al., 2011). Methylation data were examined using Kismeth software to analyze methylation at a particular site.

### Real-Time PCR Analysis

Inflorescences were collected on ice (at 4°C), florets/pistils were dissected out with the help of fine tip needles and forceps under the Zeiss streomicroscope (Stemi DV4). The dissected florets/pistils were stored in RNA Later solution (Qiagen Cat. No. 76104) for 1 day, then snap-frozen in liquid nitrogen and stored at −80°C. Total RNA was isolated from leaf, florets (FM/AI) and pistils (MES) of apomictic and sexual plants using RNeasy Plant Mini Kit (Qiagen). Synthesis of cDNA was performed with oligo-dT primers from 5 μg total RNA using BioRad iScript Select cDNA synthesis kit. To examine the effect of gene-body methylation on the expression level of integrase, RT-qPCR was performed using a pair of gypsy integrase (*Gy*163.1) specific primers (Figure 2), SYBR Green master mix (Kappa Biosystem), and the cDNA as a template on BioRad Real-time PCR machine (CFX Connect Real-time System) by 2^–ΔΔCt^ method. Estimation of relative gene expression was performed using Ct values (which are inversely related to the initial DNA concentration) for both the target and the reference (actin) gene calculated based on the mean value of three replications. Primer sequences of the reference actin gene for RT-qPCR analysis were used from a validated report of Simon et al. (2013) in which activity of various reference genes in *C. ciliaris* was reported.

## Results

### Characterization of *Gy*163 Insert in ASGR

Insertion characterization of *Gy*163 in different tissues [leaf, non-reproductive; functional megaspore (FM in a sexual plant) or aposporous initial (AI in an apomictic plant) and mature embryo sac (MES), reproductive tissues] of sexual and apomictic plants of *C. ciliaris* using RJ primers revealed its polymorphic nature between apomictic and sexual plants (Figure 3A). Primers designed for the conserved region of the *Gy*163 produced the amplicon in both sexual and apomictic individuals (Figure 3B). Protein domain analysis of the conserved monomorphic region (*Gy*163) revealed that it encodes for an integrase enzyme of RE having a catalytic (D–D-E) motif (Figure 2). In addition to this, *Gy*163 has chromodomain downstream to the integrase domain which is specific to Ty3-*gypsy* retroelements (Metaviridae). Parts (*Gy*163.1 and *Gy*163.2) of this monomorphic region representing the integrase domain were utilized to analyze the methylation status of *Gy*163 in sexual and apomictic plants.

### Methylation Level of *Gy*163 Across the Mode of Reproduction

**FIGURE 4:**
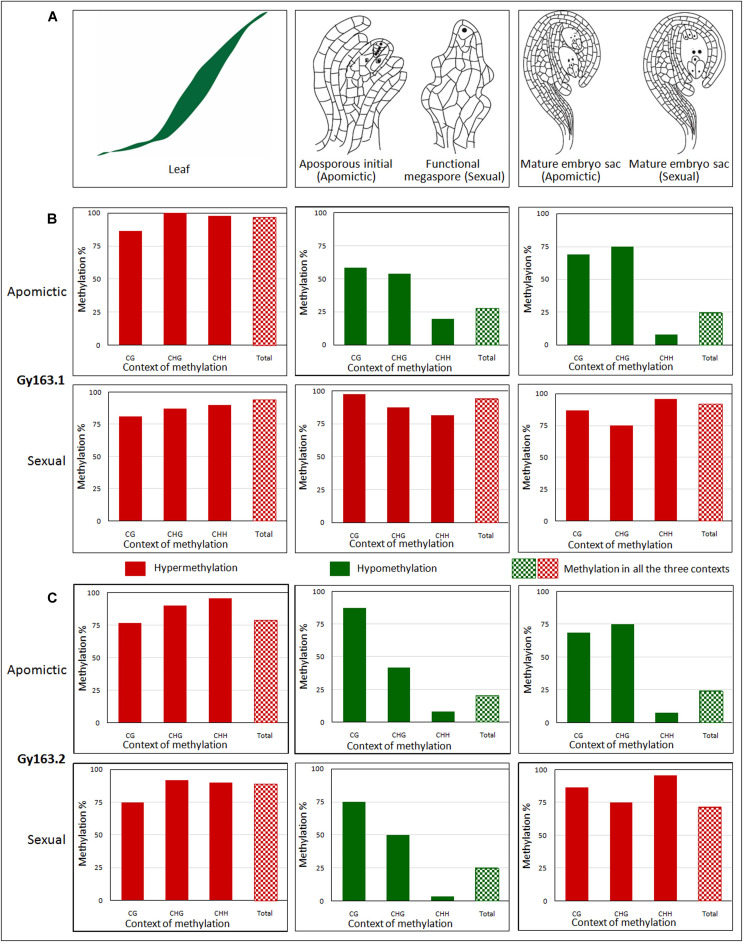
Methylation level of *Gy*163 at different contexts (CG, CHG, CHH) of cytosine in non-reproductive (leaf) and reproductive tissues [aposporous initial (AI, in apomictic), functional megaspore (FM, in sexual), and mature embryo sac (MES, in both apomictic and sexual)]. **(A)** Diagrammatic representation of (i) non-reproductive (leaf) and reproductive tissues [(ii) aposporous initial in the apomictic plant, functional megaspore in the sexual plant, and (iii) mature embryo sac in both apomictic and sexual plants]. **(B)** The methylation level of *Gy*163.1 in different tissues (Leaf, AI/FM, MES) of apomictic and sexual plants. **(C)** The methylation level of *Gy*163.2 in different tissues (Leaf, AI/FM, MES) of apomictic and sexual plants. The green bar indicates hypomethylation, while the red bar indicates hypermethylation. Bar with checkerboard (green and red) indicates total methylation at all the context of cytosine.

The methylation level of the RE *Gy*163 was analyzed in non-reproductive (leaf) and reproductive tissues [AI (in apomictic), FM (in sexual), and MES (in both apomictic and sexual plants)] (Figures 4Ai–iii). The parts (*Gy*163.1 and *Gy*163.2) of RE conserved in both apomictic and sexual, and encoding for an integrase was analyzed to investigate the association between methylation status of the RE and the mode (apomictic and sexual) of reproduction in *C. ciliaris*.

#### Methylation of *Gy*163.1 in Different Tissues of Apomictic and Sexual Plants

Cytosine methylation of *Gy*163.1 varied significantly between the non-reproductive (leaf) and reproductive (AI/FM and MES) tissues of female gametophyte development in apomictic and sexual plants. A very high level of methylation (hypermethylation) was observed in the leaf of both apomictic and sexual plants (Figures 4Bi,iv). While the methylation level in reproductive (AI and MES) tissues of the apomictic plant was low (hypomethylation) (Figures 4Bii,iii), it was very high in reproductive (FM and MES) tissues of sexual plants (Figures 4Bv,vi). Total methylation percentage in non-reproductive tissue (leaf) of apomictic and sexual plants varied between 94 and 96%. It was about 25% in the reproductive (AI and MES) tissues of apomictic plants. On the contrary, it was about 92% in the reproductive (FM and MES) tissues of sexual plants (Figures 4Bv,vi). Only a minor variation (2-3% reduction) in total methylation was observed between the early reproductive (AI/FM) tissue and advance (MES) reproductive tissues.

**FIGURE 5:**
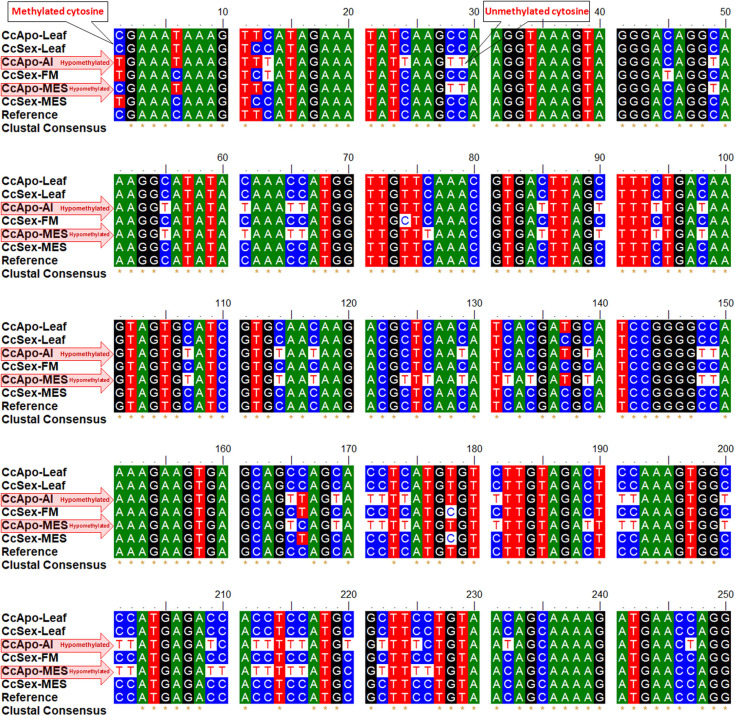
Alignment of *Gy*163.1 sequences from non-reproductive (leaf) and reproductive tissues [aposporous initial (AI, in the apomictic plant) or functional megaspore (FM, in the sexual plant), and mature embryo sac (MES, in both apomictic and sexual plants)]. Many unmethylated cytosines (T in bisulfite modified sequence, at the place of C in the reference sequence) are present in the reproductive tissues (AI and MES) of the apomictic plant.

Many of the cytosines, particularly in the CHH context, were observed to be unmethylated (present as T in the bisulfite-modified sequences, at the place of C in the reference sequence) in the reproductive tissues (AI, CcApo-AI; MES, CcApo-MES) of an apomictic plant (Figure 5). Bisulfite treatment of DNA converts unmethylated cytosine to uracil, leaving 5-mC unmodified, which gets replaced by T in due course of PCR amplification of the bisulfite modified template DNA (Kumar et al., 2017a). While most of the cytosines present in the coding region of the integrase (*Gy*163.1) gene in the vegetative tissues (leaf) of both apomictic and sexual, and reproductive tissues (FM and MES) of sexual plants were methylated (5-mC), they were unmethylated in the reproductive tissues (AI and MES) of apomictic plants.

#### Methylation Level of *Gy*163.1 at Different Contexts of Cytosine

Since the fragment of DNA analyzed in this study was a retroelement, the maximum change in methylation was observed in the CHH context, particularly in the reproductive tissues. Although a minor variation in DNA methylation was observed in the non-reproductive (leaf) tissue of apomictic and sexual plants, the maximum change was observed in CHG and CHH contexts. While analyzing methylation status in the reproductive tissues, it was observed that hypomethylation at the CHH context of *Gy*163.1 was correlated with the apomictic mode of reproduction. Methylation at all three cytosine (CG, CHG, and CHH) contexts in sexual plants was very high, particularly in the reproductive tissues (Figures 4Bv,vi).

#### Methylation of *Gy*163.2 in Different Tissues of Apomictic and Sexual Plants

*Gy*163.2 region was also observed to be hypermethylated in the non-reproductive (leaf) tissue of both apomictic and sexual plants. However, reproductive tissues (AI and MES) were observed to be hypomethylated with <25% total methylation in apomictic plants (Figure 4C). Methylation status of *Gy*163.2 was initially observed to be lower in FM (reproductive tissue) of the sexual plant (Figure 4Cv); nevertheless, it became hypermethylated in the advanced stage (MES) of reproductive development (Figure 4Cvi). Thus, the methylation status of *Gy*163.2 (Figure 4Cv) was different from that of *Gy*163.1 which is hypermethylated from the beginning (FM) (Figure 4Bv) in the sexual plants.

#### Methylation Level of *Gy*163.2 at Different Contexts of Cytosine

The methylation level of *Gy*163.2 in all the context of cytosine (CG, CHG, CHH) was observed to be very high in the leaf of apomictic and sexual plants (Figures 4Ci,iv). The maximum change in methylation was again observed in the CHH context in reproductive tissues (AI and MES) of the apomictic plants while maintaining total methylation <25% (Figures 4Cii,iii). However, the methylation status of this region in the reproductive tissue, particularly that of the FM (Figure 4Cv), did not show the correlation with the mode of reproduction, as observed in the case of *Gy*163.1 (Figure 4Bv). Except for the minor variations, methylation level in different contexts of cytosine in MES of sexual plants was observed to be similar in both *Gy*163.1 and *Gy*163.2 (Figures 4Bvi,Cvi).

### Expression Analysis of *Gy*163.1

**FIGURE 6:**
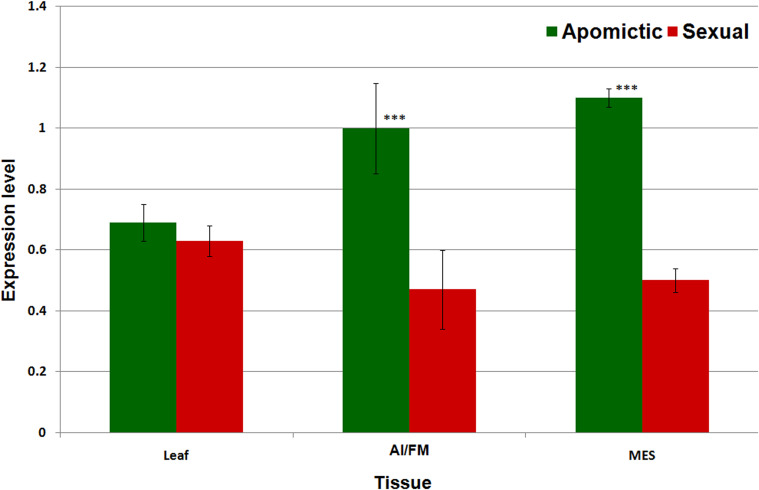
Expression profile of *Gy*163.1 in different tissues [leaf, non-reproductive tissue; aposporous initial (AI in apomictic) or functional megaspore (FM in the sexual plant) and mature embryo sac (MES in both apomictic and sexual), reproductive tissues]. Asterisks indicate significant differences (Student’s *t*-test) between the sexual and apomictic plants. ***P < 0.01.

Real-time PCR-based expression analysis of a conserved, common region of the RE *Gy*163 (encoding integrase) in the reproductive (AI/FM and MES) tissues of apomictic and sexual plants revealed that *Gy*163 was overexpressed in apomictic plants compared to that in the sexual plants (Figure 6). Comparative analysis of the expression of integrase in non-reproductive (leaf) and reproductive (AI/FM and MES) tissues indicated that expression level of the gene was 0.7 in leaf (non-reproductive tissue) of apomictic plants compared to 0.6 in the leaf of sexual plants. On the other hand, the expression level of integrase was >1.0 in reproductive (AI and MES) tissues of apomictic plants compared to ∼0.5 in the reproductive tissues (FM and MES) of sexual plants (Figure 6). An inverse correlation between the methylation level in the coding region of the integrase and the expression level of the gene could be observed. As expected, overexpression of *Gy*163.1 in the reproductive tissues (AI and MES) was associated with the apomictic mode of reproduction in the plants.

## Discussion

Apomixis has become an attractive trait to produce genetically uniform populations, and it is highly desired for crop improvement (Toenniessen, 2001; Kumar, 2017). In *C. ciliaris*, apomixis is controlled by ASGR which is around 50 Mb in size and rich in REs. Genetics of apomixis is highly complex due to uncoupled developmental events of apomeiosis and parthenogenesis (Brukhin, 2017). Several genes have been isolated and characterized but even a single regulatory mechanism controlling apomixis is not yet elucidated. Meanwhile, several studies on the epigenetic regulation of apomixis have been reported and suggested the importance of DNA methylation (Rodríguez-Leal et al., 2015). The reports suggest that methylation of genes including REs plays an important role in the origin of AI for the development of unreduced multiple embryo sacs. Mutant analyses of AGO9 in *Arabidopsis* and maize showed that the transition from somatic cell fate to reproductive fate in ovule is epigenetically controlled through siRNA-mediated DNA methylation pathway (Olmedo-Monfil et al., 2010; Singh et al., 2011). In our earlier study, several retrotransposons showing polymorphism between apomictic and sexual plants of *C. ciliaris* indicated their role in apomixis (Yadav, 2011). Hence, a detailed analysis of one of the polymorphic REs *Gy*163 was undertaken to investigate the role of epigenetic changes in controlling RE gene expression and its association with the apomictic mode of reproduction.

Insertion characterization of RE *Gy*163 using RJ primers confirmed its polymorphic nature in the apomictic and sexual plants of *C. ciliaris*. This RE was earlier reported to be polymorphic in nature and associated with the apomictic mode of reproduction in buffelgrass (Yadav, 2011). Herein, a portion of *Gy*163 was identified to be present in both apomictic and sexual plants, which encodes for the integrase enzyme involved in the movement of RE. RE activity has been suggested to be controlled through epigenetic mechanisms (Schmid et al., 2012). Therefore, we decided to analyze the methylation status of *Gy*163, and examine its role in controlling the expression of integrase in reproductive and non-reproductive tissues of apomictic and sexual plants of *C. ciliaris*. Homology based sequence analysis showed that *Gy*163contains a chromodomain downstream to the integrase gene which is a characteristic feature of some Ty3-*gypsy* integrase responsible for site-specific integration. In this study, it was hypothesized that *Gy*163 might be specifically targeted to be inserted into the ASGR of the genome with the help of chromodomain.

We observed differential methylation of *Gy*163 in vegetative (leaf) and reproductive (AI/FM and MES) tissues of apomictic and sexual plants. Hypomethylation of *Gy*163 in reproductive tissues (AI and MES) of apomictic plants and hypermethylation in FM and MES of sexual plants was observed, particularly in the CHH context. However, hypermethylation of the coding region of *Gy*163 was observed in the leaf tissue of both apomictic and sexual plants. *Gy*163.1 was hypermethylated (upto 94%) in all the tissues (leaf, FM, and MES) of sexual plants, while it was hypomethylated (∼25%) in AI and MES of apomictic plants. However, the methylation level of Gy163 in the sexual plants was observed to vary between *Gy*163.1 and *Gy*163.2, particularly in the functional megaspore (FM) tissues. *Gy*163.1 and *Gy*163.2 (approx. 440 bp each) are the segments of the coding region of an integrase gene, and the methylation level of the *Gy*163.1 was observed in this study to regulate the expression level of the gene. This finding corroborates with the earlier reports on variation in methylation level of different parts (e.g., 5′ and 3′) of the coding region (Jones, 2012). Hence, the variation in the methylation level of *Gy*163.1 and *Gy*163.2 is normal and interesting as it indicates the role of gene-body methylation in the regulation of gene expression. Hypermethylation in the coding region causes decreased expression of the integrase in different tissues of the sexual plants. On the other hand, hypomethylation of the coding region of integrase (*Gy*163.1 and *Gy*163.2) caused the higher expression of the gene in AI and MES tissues of apomictic plants. Previous reports on REs indicate their differential expression in apomictic and sexual plants (Dolgin and Charlesworth, 2006; Ochogavıa et al., 2011; Podio et al., 2014; Zappacosta et al., 2014; Shimada et al., 2018), which corroborate our findings.

No significant difference in the expression level of *Gy*163 at AI and MES stages of female gametophyte development in apomictic plants. Therefore, our study indicated that *Gy*163 might play a role in female gametophyte development from the differentiation of aposporous initial to unreduced embryo sac development.

No major difference in methylation of *Gy*163 in leaf (vegetative) of apomictic and sexual plants was expected because this organ does not play a role in the reproduction process. *Gy*163, being a retrotransposon, showed a considerable change in cytosine methylation in the CHH context. Methylation in asymmetric (CHH) context is performed by Domain Rearranged Methyltransferase1 (DRM1) and DRM2 through RNA-directed DNA methylation (RdDM) pathway, which inactivates REs and silences the target gene (Law and Jacobsen, 2010; Zhang et al., 2010). Jullien et al. (2012) reported enhanced expression of DRM1 and activated RdDM pathway in the egg cell which resulted in the silencing of retrotransposons in the female germline. Thus, cytosine (de) methylation in the CHH context plays an important role in retrotransposon activity and may decide the apomictic or sexual mode of reproduction in the plant.

Differential expression of DNA methyltransferase (DMTs) in apomictic and sexual plants and *dmts* mutants showing the development of multiple unreduced embryo sacs have been reported earlier (Grimanelli, 2012). In *Arabidopsis thaliana*, reduced RE activity was reported in *ago9* mutants resulting in the ectopic development of unreduced female gametophyte (Olmedo-Monfil et al., 2010). Single-cell studies onthe distribution of the enzymes and other protein complexes responsible for asymmetric methylation controlling the female gametophyte development may reveal the epigenetic basis of apomixis. Epigenetic modification helps to adapt a plant to changed environmental conditions, and this may also play a role in setting an appropriate mode of reproduction according to the environment.

In conclusion, our findings indicate that hypomethylation of *Gypsy*163 in the apomictic plant might be responsible for the initiation of apomictic seed development in *C. ciliaris*. Demethylation of the coding region of *Gy*163 in CHH context may be responsible for enhanced expression of integrase in the female gametophyte; thus, the activity of the RE, and initiates apomictic seed development. This also suggests that activation of *Gy*163 and its insertion in ASGR is associated with the apomictic mode of reproduction in *C. ciliaris*.

## Data Availability Statement

The datasets presented in this study can be found in online repositories. The names of the repository/repositories and accession number(s) can be found in the article/supplementary material.

## Author Contributions

PR, VB, and SK planned and designed the experiments. PR carried out the experiments. PR, VB, SR, and SK analyzed the data, prepared/revised the manuscript, and approved the final draft. All authors contributed to the article and approved the submitted version.

## Conflict of Interest

The authors declare that the research was conducted in the absence of any commercial or financial relationships that could be construed as a potential conflict of interest.

## Footnotes

1 https://probes.pw.usda.gov/RJPrimers/

2 http://www.repeatmasker.org/

3 http://repeatexplorer.org/
